# Supplementary material for: Autotrophic Acetate Production under Hydrogenophilic and Bioelectrochemical Conditions with a Thermally Treated Mixed Culture
Source: Membranes (Basel). 2022 Jan 21;12(2):126. doi: 10.3390/membranes12020126 (PMC8876840; doi:10.3390/membranes12020126)
Supplement: Supplementary file 1 [file membranes-12-00126-s001.zip › membranes-1553474-supplementary.pdf]

membranes

Article

Thermal treated mixed culture under hydrogenophilic and bio-electrochemical condition for acetate autotrophic production

Jacopo Ferretti, Lorenzo Cristiani, Marianna Villano, Marco Zeppilli*

Department of Chemistry, Sapienza University of Rome, P.le Aldo Moro 5, 00185 Rome, Italy
* Correspondence: marco.zeppilli@uniroma1.it

Supplementary material

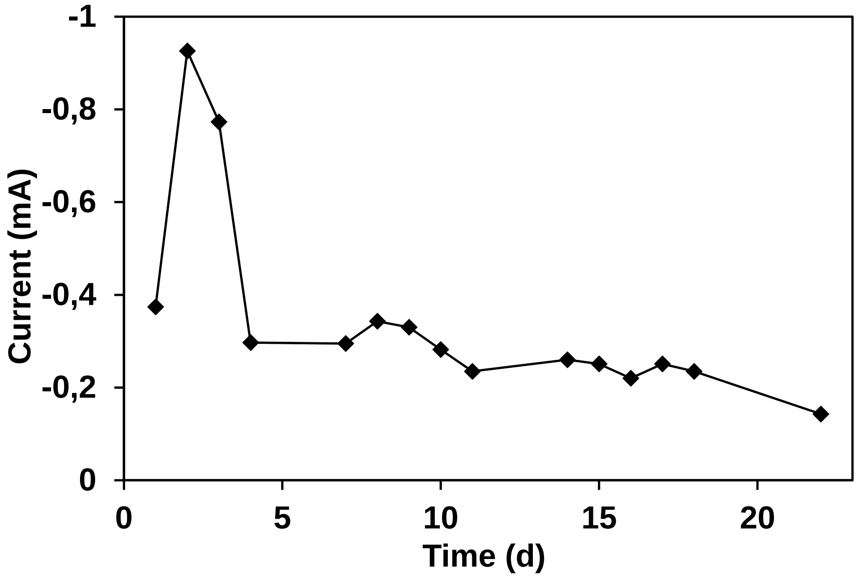

Figure S1. Time profile of the current generated in the bioelectrochemical batch cell.

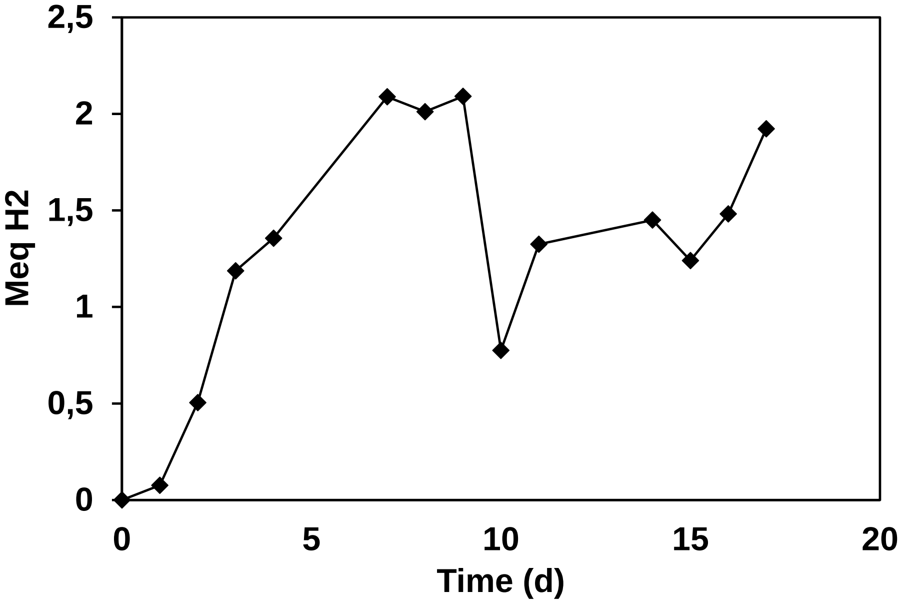

Figure S2. Time profile of the hydrogen in the cathodic chamber of the bioelectrochemical batch cell.

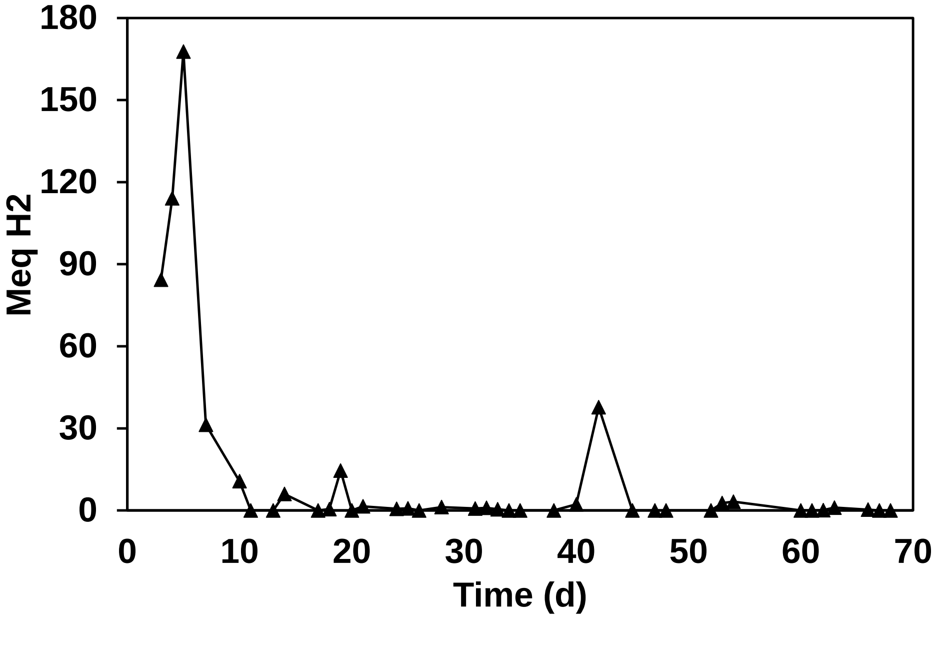

Figure S3. Time profile of the hydrogen produced in the continuous flow bioelectrochemical reactor.

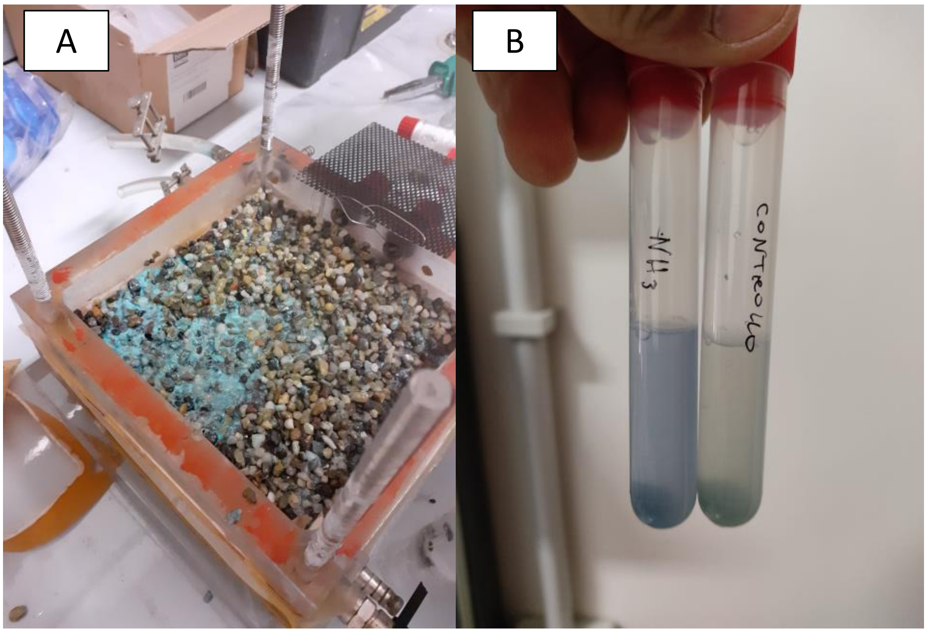

Figure S4. Insoluble precipitate on the FAD membrane (A), qualitative test for copper determination (B)

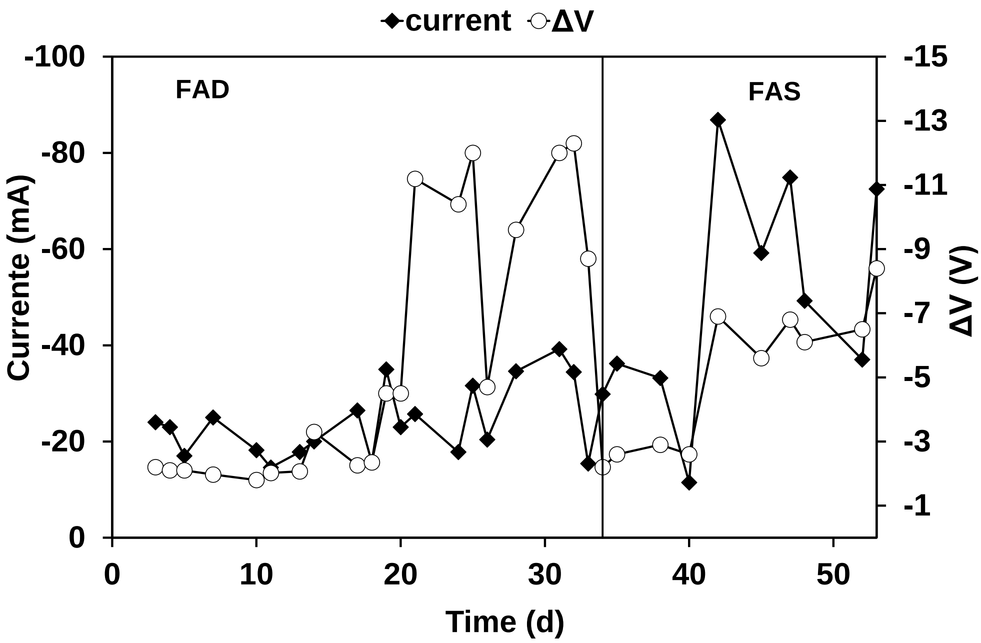

Figure S5. Time profile of current (mA) and cell voltage (V) in the continuous flow bioelectrochemical reactor.
